# Which protocol reflects more reliable facts about learning and memory in fruit flies?

**DOI:** 10.17179/excli2019-1376

**Published:** 2019-07-12

**Authors:** Mohammad Haddadi

**Affiliations:** 1 Department of Biology, University of Zabol, Zabol, Iran

## ⁯

***Dear editor,***

An urgent need for better understanding of memory is increasing as our aged population gets older and older. Extensive research on the biology of learning, memory and neurodegeneration is accelerating to meet this challenge. *Drosophila melanogaster* is shown to play an essential role in unraveling the genetics of learning and memory. Various behavioral assays on *Drosophila* model of human neurodegenerative diseases have been employed in a number of studies. Classical olfactory conditioning, courtship conditioning, and newly-introduced method based on predator-mediated fear induction are among the most widely applicable and reproducible ones. In this letter I wish to address some critical aspects of each protocol, though the given points can be considered as a general criticism for such kinds of experiments.

The classical olfactory conditioning pairs a conditioned stimulus such as an odor with an unconditioned (training-free) stimulus such as a painful electric shock, heat shock, or starvation. Usually two aversive chemicals are used in each experimental setup. One odor is associated with a painful electric shock, while the other is not. Memory of this conditioning is subsequently evaluated by investigating the fly preference between the two scents. Fly avoidance of the electric shock-paired odor is the basis for quantifying the performance indices (Busto et al., 2010[1]). This procedure uses both male and female flies. Although the stimuli are not natural and flies never expose to them during their normal life in nature, both sexes are examined in the same protocol. Moreover, the same protocol can be applied for the larvae in order to assess neural function during developmental stages.

In courtship conditioning if a male fly has been rejected several times by a mated female fly, it eventually fails to send mating signals to the female, even if next time a virgin female fly is encountered. As a consequence of past fly encounters, the mating avoidance behavior is memorized. The time which is taken by male fly to initiate reproduction behavior is termed the performance index (Kamyshev et al., 1999[3]). Though the protocol uses natural cues with no man-made artificial components, it is only based on the male fly behavior. Female flies are neither involved in learning and memory formation nor in calculation of performance index.

The fear conditioning protocol employs a *Drosophila* predator, such as a wasp, to provoke conventional defense behaviors (Kacsoh et al., 2015[2]). In this protocol, a female fly is exposed to the predator for a short period of time and then oviposition behavior of the fly is observed following the predator removal. Visual signals produced by predator exposure can induce stress and result in a change in the fly egg-laying behavior. In this learning and memory paradigm only females are tested and the male response to predator is not generally examined.

Understanding memory in both genders is important to our society. Therefore, it would seem prudent to use a versatile sex-bias free behavioral test. Classical olfactory conditioning experiments offer many advantages where seeking the result of a genetic mutation, environmental factors, or neurotoxic agents.
